# Effects of SGLT-2 inhibitors and GLP-1 receptor agonists on liver function in patients with non-alcoholic fatty liver disease and type 2 diabetes

**DOI:** 10.3389/fendo.2026.1865769

**Published:** 2026-06-03

**Authors:** Wancheng Guo, Wenhe Li, Xianlin Li, Wen Shi, Yan Yan, Ting Yan, Jie Zhou, Yujie Huang

**Affiliations:** 1 Department of Endocrinology, Quanjiao County People’s Hospital, Chuzhou, China; 2 Department of Endocrinology, Huai 'an Clinical Medical College of Jiangsu University, Huaian, China; 3 Department of Endocrinology, Huai 'an Hospital of Huai 'an City, Huaian, China

**Keywords:** clinical trial, glucagon-like peptide-1 receptor agonists, non-alcoholic fatty liver disease, sodium-glucose cotransporter-2 inhibitors, type 2 diabetes

## Abstract

**Background:**

The goal was having the comparisons be direct when it came to seeing how much of a help the sodium-glucose co transporter - 2 inhibitors (SGTL2is) compared to the glucagon-like peptide-1 receptor agonists (GLP-1RAs) were in regard to how the liver enzymes performed within people having type 2 diabetes (T2D) along with nonalcoholic fatty liver disease (NAFLD), a condition characterized by excessive fat accumulation in the liver without alcohol overconsumption.

**Methods:**

A total of 705 subjects with T2DM and NAFLD at time of first SGLT2is (n=381) or GLP-1RAs (n=324) enrolment in this multicentre posterior cohort study from 01/2020-12/2024. Baseline characteristics were made equivalent via PSM via a 1: 1 NN match and a 0. 2 SD match clamp. The main measurement was the difference in alanine amino transferase (ΔALT) and aspartate aminotransferase (ΔAST) from the beginning and 6 months. And other result was about the change in metabolic parametres. The independent predictors of liver-enzyme change came from the multivariate linear regression analysis.

**Results:**

After PSM, 243 well-matched pairs were successfully identified, with baseline characteristics acceptably balanced (standardized differences <0.2 for all covariates and <0.1 for most covariates). In the matched cohort, SGLT2is treatment was associated with significantly greater reductions in ALT (ΔALT: -10.55 ± 12.66 vs. -7.28 ± 15.34 U/L, p=0.011) and AST (ΔAST: -7.68 ± 10.07 vs. -5.18 ± 11.04 U/L, p=0.010) compared to GLP-1RAs treatment. No significant differences were observed for changes in GGT, body weight, glycemic control, or lipid profiles between groups. Multivariable regression analysis revealed that SGLT2is treatment was independently associated with reductions in both ALT (β = -3.34, p=0.009) and AST (β = -2.32, p=0.016). Weight change was independently associated with AST reduction (β = 0.22, p=0.016) but not with ALT reduction.

**Conclusion:**

SGLT2is were associated with greater ALT and AST reductions than GLP-1RAs in T2D patients with NAFLD, with ALT improvement independent of weight loss, suggesting potential direct hepatoprotective effects.

## 1 Introduction

The prevalence of non-alcoholic fatty liver disease (NAFLD) is rising globally, affecting approximately 25% of adults worldwide (1). It is a metabolic disorder closely associated with insulin resistance (2). Likewise, diabetes is also common around the world. According to the latest report by the International Diabetes Federation, 10.5% of the adult population is affected by it. NAFLD occurs a lot more in individuals who have type 2 diabetes (T2D). Approximately 60–80% of T2D patients present with NAFLD or advanced liver disease (3, 4). Conversely, the presence of NAFLD increases the incidence of T2D and accelerates the progression of its complications (5). This coexistence accelerates the progression of liver disease, from simple steatosis to steatohepatitis, fibrosis, and cirrhosis. Furthermore, the combination of NAFLD and T2D is associated with a substantially increased risk of cardiovascular events and all-cause mortality compared with either condition alone (6, 7). Consequently, actively managing coexisting NAFLD has become a critical strategy for improving long-term patient outcomes in T2D management (8).

In recent years, novel hypoglycaemic agents represented by glucagon-like peptide-1 receptor agonists (GLP-1RAs) and sodium-glucose cotransporter-2 inhibitors (SGLT2is) have been reported not only to exhibit significant blood glucose-lowering effects but also to confer cardiorenal protective benefits (9, 10). Furthermore, evidence suggests these medications may directly improve hepatic steatosis by upregulating insulin signalling pathways (11). The accumulating evidence for the efficacy of both classes of drugs in NAFLD offers compelling therapeutic options for affected patients (12, 13). However, although both produce hepatoprotective effects, their mechanisms of action differ fundamentally. GLP-1RAs exert their hepatoprotective effects primarily via indirect pathways, namely central appetite suppression, weight loss, and improvements in systemic metabolism and inflammatory status (14, 15); whereas SGLT2Is exert a more direct effect on the liver through dual direct and indirect mechanisms, including energy metabolism reprogramming, mitochondrial protection, and restoration of intracellular calcium homeostasis (16). Although numerous studies have separately examined the improvement in hepatic outcomes of GLP-1RAs or SGLT2is compared to placebo or conventional hypoglycaemic agents, research directly comparing the hepatoprotective effects of these two cutting-edge drug classes in patients with T2D and NAFLD remains scarce (17). It remains unclear whether the two drug classes, owing to their differing mechanisms of action, will exert distinct patterns of influence on liver-specific injury markers (such as alanine aminotransferase [ALT] and aspartate aminotransferase [AST]), nor is it known whether there are differences between them in terms of hepatoprotective benefits (18).

Against this background, this study designed a multicentre retrospective cohort study to directly compare the effects of GLP-1RAs and SGLT2is on hepatic biochemical markers (primarily ALT and AST) in patients with T2D complicated by NAFLD. The aim was to investigate whether there are differences in their hepatoprotective effects, thereby providing direct comparative evidence to assist clinicians in selecting hypoglycaemic regimens that also offer liver protection.

## 2 Materials and methods

### 2.1 Study design and data source

This is a multicentre, retrospective cohort Study. Data comes from people seeing the Endocrinologist at our hospital between January 2020 and December 2024. And it’s got the nod from the local ethics board (HAYY-IRB-KY-2026-001), plus it stays true to the revised Helsinki declaration. As this is a retrospective study no need of informed consent. The trial has been registered on clinicaltrials. gov (ChiCTR2600121908).

### 2.2 Study participants

We identified all adults (aged ≥20 years) with NAFLD and T2D who initiated continuous monotherapy with GLP-1RAs or SGLT2is for at least six months. The study cohort was restricted to patients with at least one year of follow-up prior to inclusion (i.e., the time between the first prescription for glucose-lowering medication and cohort entry exceeded one year).

NAFLD is first discovered through special ICD-9 and ICD-10 code. In the NAFLD group, other patients who were diagnosed with some other liver diseases (ICD code) were excluded. Exclusion criteria: liver disease that is not caused by alcohol, severe hepatic failure, cirrhosis, hepatocellular carcinoma, concomitant severe systemic disease, pregnant or lactating, switching to another drug during this study, missing a key.

### 2.3 Identification of NAFLD

Patients with NAFLD were identified from the electronic health record database using the International Classification of Diseases codes. The case definition encompassed ICD-9-CM codes 571.8 (other chronic nonalcoholic liver disease) and 571.9 (unspecified chronic liver disease without mention of alcohol), as well as ICD-10-CM codes K76.0 (fatty liver, not elsewhere classified), K75.81 (nonalcoholic steatohepatitis, NASH), and K76.89 (other specified diseases of the liver). To ensure diagnostic specificity, patients with codified diagnoses of alcoholic liver disease (ICD-9-CM: 571.0–571.3; ICD-10-CM: K70.0–K70.9), chronic viral hepatitis B or C (ICD-9-CM: 070.2, 070.3, 571.4; ICD-10-CM: B16.x, B18.0–B18.2), autoimmune hepatitis (ICD-10-CM: K75.4), Wilson disease (ICD-10-CM: E83.0), and hemochromatosis (ICD-10-CM: E83.11) were excluded from the study cohort.

### 2.4 Data collection

All clinical information for this study was sourced from the centre’s existing medical record system. The research team retrospectively extracted clinical data from patients who received GLP-1RAs or SGLT2is therapy at the centre between January 2020 and December 2024 and met inclusion criteria. This was conducted by trained researchers following a predefined standardised data extraction protocol.

Baseline data for all eligible patients were collected for each indicator within the three months preceding drug initiation, alongside relevant indicators following six months of continuous drug treatment. Data collection primarily encompassed the following dimensions: demographic and clinical baseline information (age, gender, height, weight, body mass index, waist circumference, and relevant comorbidities); laboratory parametres (liver function, glucose metabolism, lipid profile, serum uric acid, and FIB-4 index); and detailed medication information (drug type, initiation date, dosage, and adjustment history).

### 2.5 Biochemical testing

All laboratory parameters were assessed at baseline and at the 6-month follow-up, including liver enzymes (ALT, AST, GGT), glucose metabolism markers and lipid profile parameters, all of which were measured using a standard fully automated analyser (Beckman Coulter AU5800). The reference ranges are: ALT 10–40 U/L, AST 10–40 U/L and GGT 10–60 U/L for adults. Glycated haemoglobin (HbA1c) is measured using high-performance liquid chromatography (reference range 4.0–6.0%).

### 2.6 Outcome

Primary endpoints included changes in liver function (AST, ALT and GGT) from baseline to six months (ΔALT, ΔAST and ΔGGT). Secondary endpoints comprised changes in metabolic parameters, body weight, waist circumference, and other relevant indicators.

### 2.7 Statistical analysis

All statistical analyses in this study were performed using R software (version 4.3.2). Continuous variables that met normal distribution were expressed as mean ± standard deviation; those that did not were presented as median (interquartile range). Categorical variables were reported as frequency (percentage). All statistical tests were two-tailed, with P < 0.05 considered statistically significant.

We employed propensity score matching (PSM) to control for baseline confounders and enhance comparability between patient cohorts. Specifically, we compared patients receiving GLP-1RAs therapy with those receiving SGLT2is therapy. Prior to matching, we first constructed a logistic regression model with treatment group (SGLT2is vs. GLP-1RAs) as the dependent variable and baseline variables with significant differences as covariates to calculate each patient’s propensity score for receiving SGLT2is therapy. The covariates included in the model were: age, sex, body mass index, fasting blood glucose, glycated haemoglobin, liver function markers (alanine aminotransferase, aspartate aminotransferase, gamma-glutamyltransferase), and lipid markers (total cholesterol, triglycerides, high-density lipoprotein cholesterol, low-density lipoprotein cholesterol).

Subsequently, a 1:1 PSM was conducted using the nearest neighbour approach. To ensure the quality of the matched pairs, calipers were set at a width of 0.2 times the standard deviation of the logit propensity score. Post-matching, we assessed intergroup balance by calculating standardized mean differences across all covariates. An absolute SMD <0.10 was considered excellent balance, while SMDs between 0.10 and 0.20 were considered acceptable with minor residual imbalance. All subsequent efficacy analyses were conducted on the matched cohort.

Comparisons between groups for primary outcome measures (ΔALT, ΔAST and ΔGGT) and secondary outcome measures (including ΔFIB-4, and changes in metabolic parametres) were performed using independent samples t-tests. To further evaluate the independent effect of the treatment drug, two multivariate linear regression models were constructed: one with ΔALT and another with ΔAST as the dependent variable. Independent variables included treatment group, Δbody weight, ΔHbA1c, baseline corresponding liver enzyme values, age, and gender. All results are presented as effect sizes with 95% confidence intervals.

## 3 Results

### 3.1 Baseline demographics

**Figure 1:**
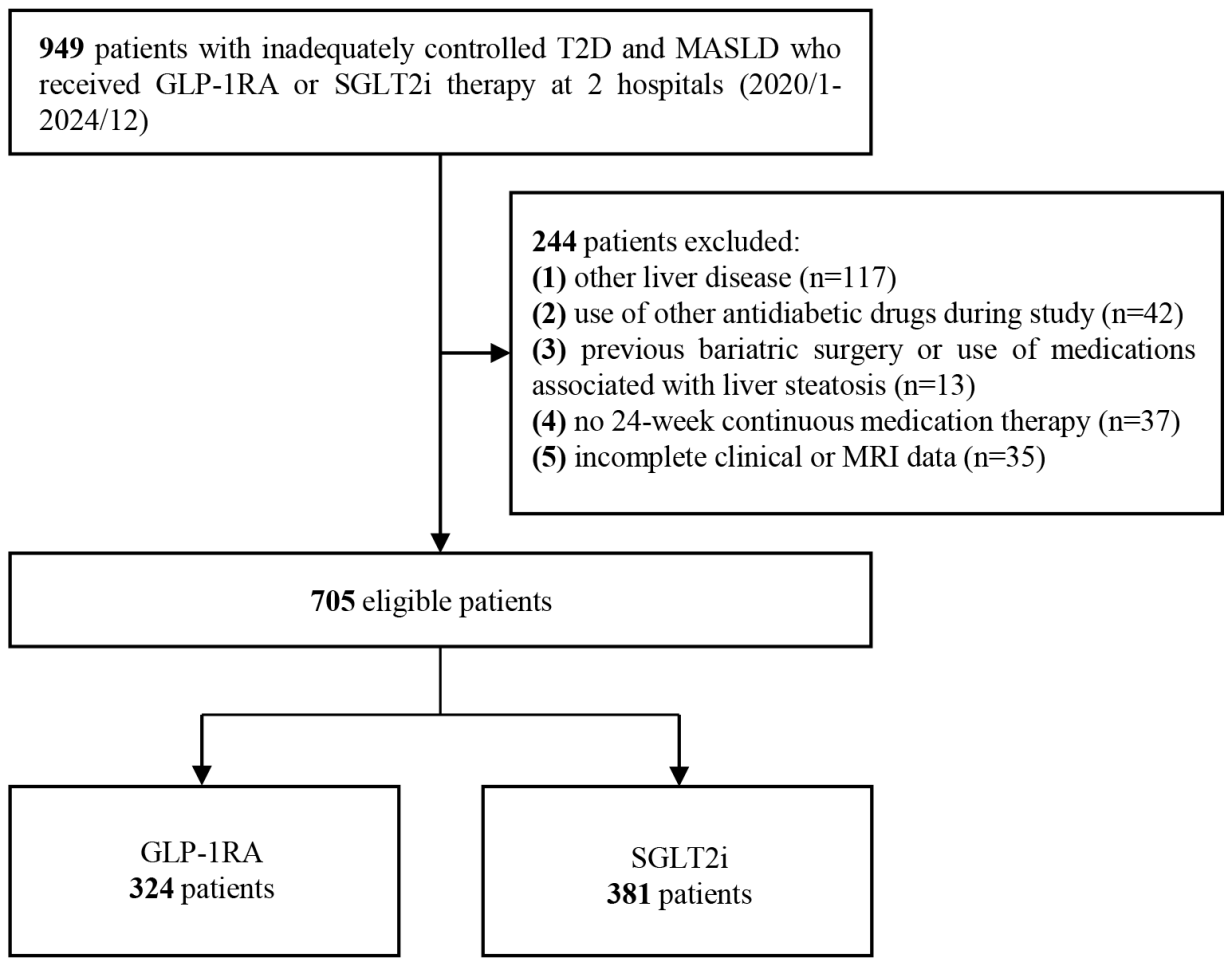
Flow chart of patient selection in the study cohort. GLP-1RA, glucagon-like peptide-1 receptor agonists; SGLT2i, sodium–glucose cotransporter 2 inhibitors; T2D, type 2 diabetes; MASLD, metabolic dysfunction-associated steatotic liver disease.

Between January 2020 and December 2024, a total of 949 patients with T2D and NAFLD who were treated with GLP-1RAs or SGLT2is were identified. After screening, a total of 705 patients were ultimately included. Based on the therapeutic agents used, the patients were divided into a GLP-1RAs group and an SGLT2is group, comprising 324 and 381 patients, respectively (**Figure 1**). Prior to PSM, there were significant differences in baseline characteristics between the two groups. Patients receiving SGLT2is treatment were younger (49.42 ± 8.64 vs. 53.98 ± 6.94, p<0.001). Patients in the GLP-1RAs group had higher levels of total cholesterol (TC), high-density lipoprotein (HDL), low-density lipoprotein (LDL), and AST. In contrast, the SGLT2i group exhibited higher levels of fasting blood glucose (FBG), triglycerides (TG), ALT, and γ-glutamyl transferase (GGT). After PSM, most baseline covariates achieved good balance with standardized differences <0.1. Body weight and AST showed minor residual imbalance (both SMD = 0.131), which remain within the acceptable range (<0.2). A total of 243 patients were retained in each group (**Table 1**).

**Table 1:** Baseline clinical characteristics of individuals with T2D and MASLD who received GLP-1RA or SGLT2i therapy.

Variables	Before PSM				After PSM			SMD
	GLP-1RA (n = 324)	SGLT2i (n= 381)	P value	SMD	GLP-1RA (n = 243)	SGLT2i (n= 243)	P value	
Age	53.98 ± 6.94	49.42 ± 8.64	<0.001*	0.582	52.28 ± 6.48	51.64 ± 7.89	0.329	0.089
Male, n (%)	190 (58.6)	236 (61.9)	0.415	0.067	148 (60.9)	146 (60.1)	0.926	0.017
Body weight (kg)	84.18 ± 13.59	84.30 ± 14.01	0.908	0.009	83.79 ± 13.58	85.57 ± 13.58	0.150	0.131
BMI (kg/m^2^)	30.58 ± 4.16	30.84 ± 5.13	0.468	0.055	30.55 ± 4.05	30.87 ± 4.95	0.442	0.070
Waist measurement (cm)	89.75 ± 7.99	90.05 ± 6.48	0.586	0.041	89.67 ± 8.03	90.14 ± 6.58	0.484	0.064
FBG (mmol/L)	7.23 ± 1.23	7.51 ± 2.08	0.034*	0.164	7.31 ± 1.24	7.25 ± 2.08	0.653	0.041
HbA1c (%)	8.09 ± 1.31	8.19 ± 0.80	0.233	0.088	8.09 ± 1.30	8.19 ± 0.79	0.299	0.094
Uric acid (umol/L)	375.21 ± 75.61	385.19 ± 85.03	0.103	0.124	377.98 ± 75.26	383.81 ± 84.66	0.423	0.073
Albumin (g/L)	47.31 ± 3.46	47.47 ± 3.25	0.520	0.048	47.36 ± 3.43	47.50 ± 3.30	0.631	0.044
TC (mmol/L)	4.79 ± 1.84	4.55 ± 1.26	0.042*	0.151	4.66 ± 1.80	4.59 ± 1.23	0.637	0.043
TG (mmol/L)	1.99 ± 0.28	2.11 ± 0.58	0.001*	0.270	2.01 ± 0.28	2.02 ± 0.56	0.709	0.034
HDL (mmol/L)	1.11 ± 0.17	1.04 ± 0.23	<0.001*	0.310	1.09 ± 0.17	1.07 ± 0.23	0.372	0.081
LDL (mmol/L)	2.73 ± 0.72	2.51 ± 0.74	<0.001*	0.302	2.62 ± 0.72	2.58 ± 0.72	0.517	0.059
ALT (U/L)	34.10 ± 14.99	37.76 ± 12.66	<0.001*	0.264	36.58 ± 15.26	36.06 ± 12.04	0.674	0.038
AST (U/L)	29.68 ± 9.73	27.08 ± 9.83	<0.001*	0.266	28.98 ± 9.89	27.72 ± 9.42	0.150	0.131
GGT (U/L)	46.98 ± 9.64	49.08 ± 11.81	0.011*	0.195	48.11 ± 9.70	48.88 ± 11.92	0.431	0.072
FIB-4 index	1.06 ± 0.33	1.08 ± 0.25	0.624	0.037	1.06 ± 0.33	1.08 ± 0.26	0.510	0.060

Data are presented as mean ± SD or n (%) for categorical variables. *Indicates statistical significance. GLP-1RA, glucagon-like peptide-1 receptor agonists; SGLT2i, sodium–glucose cotransporter 2 inhibitors; T2D, type 2 diabetes; MASLD, metabolic dysfunction-associated steatotic liver disease; BMI, body mass index; FBG, fasting blood glucose; HbA1c, haemoglobin A1c; TC, total cholesterol; TG, triglycerides; HDL, high-density lipoprotein; LDL, low-density lipoprotein; ALT, alanine aminotransferases; AST, aspartate aminotransferases; GGT, γ-glutamyl transferase; FIB-4, fibrosis-4 index.

### 3.2 Primary outcome

**Figure 2:**
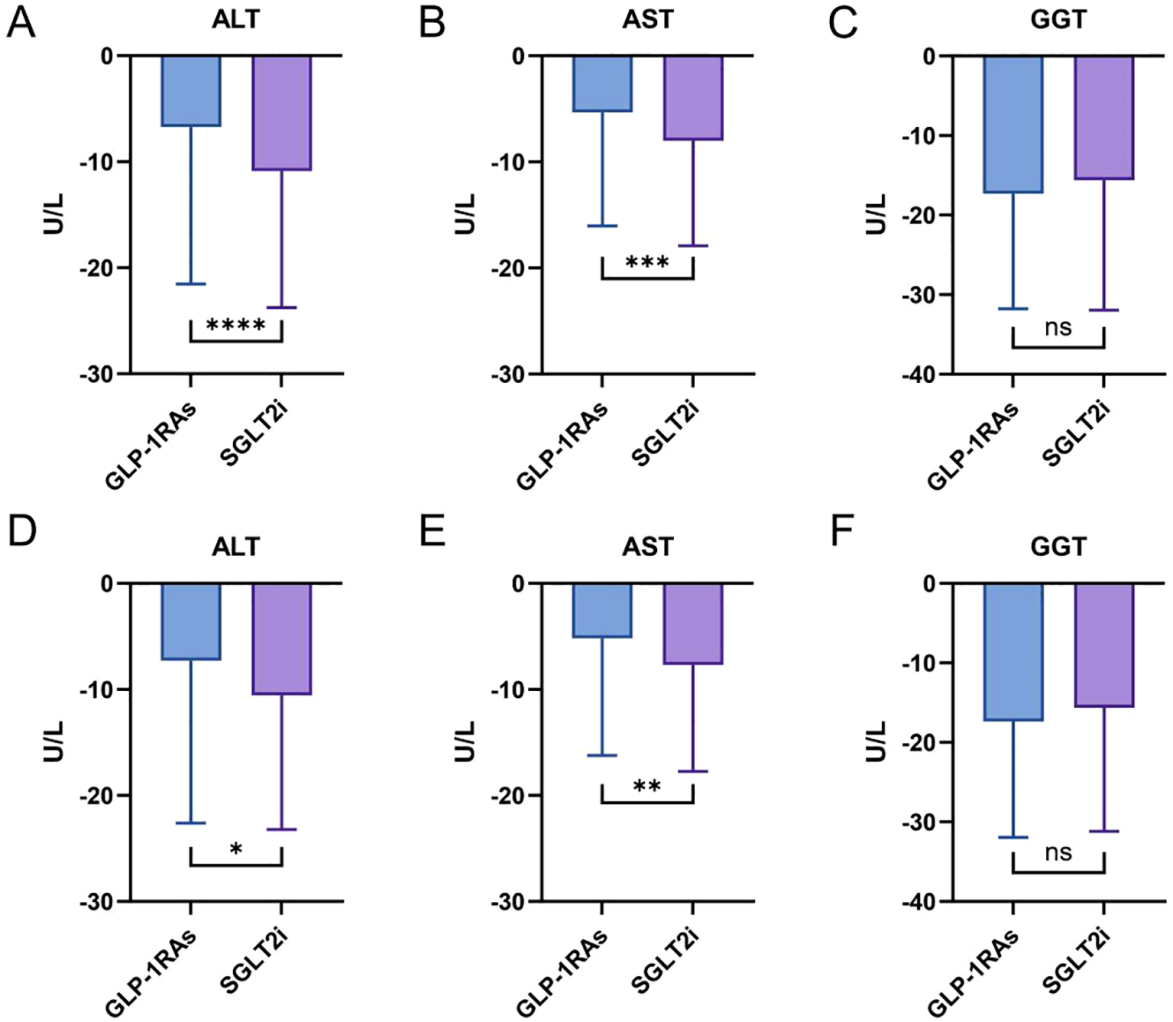
Changes in **(A)** ALT, **(B)** AST, and **(C)** GGT in patients with T2D and MASLD treated with GLP-1RA or SGLT2i, before PSM. Changes in **(D)** ALT, **(E)** AST, and **(F)** GGT after PSM. GLP-1RA, glucagon-like peptide-1 receptor agonists; SGLT2i, sodium–glucose cotransporter 2 inhibitors; T2D, type 2 diabetes; MASLD, metabolic dysfunction-associated steatotic liver disease; ALT, alanine aminotransferases; AST, aspartate aminotransferases; GGT, γ-glutamyl transferase; PSM, propensity score matching. *P < 0.05, **P < 0.01, ***P < 0.001, ****P < 0.0001.

Before matching, the SGLT2is group demonstrated significantly greater reductions in ALT (ΔALT: -10.89 ± 12.88 U/L) and AST (ΔAST: -8.02 ± 9.89 U/L) compared to the GLP-1RAs group (ΔALT: -6.73 ± 14.80 U/L; ΔAST: -5.34 ± 10.71 U/L), with all P-values < 0.01. In the matched cohort after eliminating baseline confounders, the SGLT2is group continued to show significantly larger reductions in ALT (ΔALT: -10.55 ± 12.66 U/L) and AST (ΔAST: -7.68 ± 10.07 U/L) than the GLP-1RA group (ΔALT: -7.28 ± 15.34 U/L; ΔAST: -5.18 ± 11.04 U/L), and the differences remained statistically significant (ΔALT: P = 0.011; ΔAST: P = 0.010). However, changes in GGT showed no significant difference between the two groups either before or after matching (**Table 2**, **Figure 2**).

**Table 2:** Changes in clinical characteristics of individuals with T2D and MASLD who received GLP-1RA or SGLT2i therapy.

	Before PSM			After PSM		
Variables	GLP-1RA (n = 324)	SGLT2i (n= 381)	P value	GLP-1RA (n = 243)	SGLT2i (n= 243)	P value
Δ Body weight (kg)	-5.10 ± 4.80	-5.83 ± 5.26	0.055	-5.04 ± 4.94	-5.49 ± 5.45	0.348
Δ BMI (kg/m2)	-1.96 ± 2.01	-2.18 ± 1.60	0.111	-1.84 ± 2.02	-2.13 ± 1.62	0.080
Δ Waist measurement (cm)	-5.73 ± 3.73	-5.40 ± 3.00	0.200	-5.42 ± 3.77	-5.51 ± 3.02	0.776
Δ FBG (mmol/L)	-1.03 ± 1.34	-1.30 ± 1.81	0.027*	-1.09 ± 1.31	-1.27 ± 1.81	0.206
Δ HbA1c (%)	-1.83 ± 1.11	-1.95 ± 1.14	0.162	-1.87 ± 1.15	-1.88 ± 1.14	0.922
Δ Uric acid (umol/L)	-30.86 ± 23.36	-34.13 ± 25.89	0.081	-31.50 ± 23.45	-33.80 ± 24.95	0.294
Δ Albumin (g/L)	1.35 ± 2.11	1.45 ± 2.60	0.607	1.36 ± 2.09	1.36 ± 2.74	0.992
Δ TC (mmol/L)	-0.14 ± 0.34	-0.08 ± 0.42	0.053	-0.14 ± 0.33	-0.08 ± 0.43	0.080
Δ TG (mmol/L)	-0.15 ± 0.24	-0.13 ± 0.39	0.327	-0.15 ± 0.24	-0.12 ± 0.39	0.273
Δ HDL (mmol/L)	0.05 ± 0.25	0.04 ± 0.26	0.852	0.04 ± 0.26	0.07 ± 0.25	0.260
Δ LDL (mmol/L)	-0.32 ± 0.91	-0.28 ± 0.63	0.409	-0.35 ± 0.94	-0.27 ± 0.65	0.282
Δ ALT (U/L)	-6.73 ± 14.80	-10.89 ± 12.88	<0.001*	-7.28 ± 15.34	-10.55 ± 12.66	0.011*
Δ AST (U/L)	-5.34 ± 10.71	-8.02 ± 9.89	0.001*	-5.18 ± 11.04	-7.68 ± 10.07	0.010*
Δ GGT (U/L)	-17.30 ± 14.49	-15.62 ± 16.32	0.153	-17.38 ± 14.56	-15.64 ± 15.54	0.204
Δ FIB-4 index	-0.15 ± 0.31	-0.13 ± 0.34	0.236	-0.14 ± 0.31	-0.12 ± 0.35	0.476

Data are presented as mean ± SD. *Indicates statistical significance. GLP-1RA, glucagon-like peptide-1 receptor agonists; SGLT2i, sodium–glucose cotransporter 2 inhibitors; T2D, type 2 diabetes; MASLD, metabolic dysfunction-associated steatotic liver disease; BMI, body mass index; FBG, fasting blood glucose; HbA1c, haemoglobin A1c; TC, total cholesterol; TG, triglycerides; HDL, high-density lipoprotein; LDL, low-density lipoprotein; ALT, alanine aminotransferases; AST, aspartate aminotransferases; GGT, γ-glutamyl transferase; FIB-4, fibrosis-4 index.

### 3.3 Secondary outcomes

Before adjustment, the SGLT2is group exhibited a greater reduction in FBG levels. However, this difference disappeared after adjustment. For the remaining secondary outcome measures—including body weight, BMI, waist circumference, ΔHbA1c, blood lipids (ΔTC, ΔTG, ΔHDL, ΔLDL), FIB-4, and other indices—no statistically significant differences (P > 0.05) were observed either before or after matching (**Table 2**).

### 3.4 Multiple linear regression analysis

**Figure 3:**
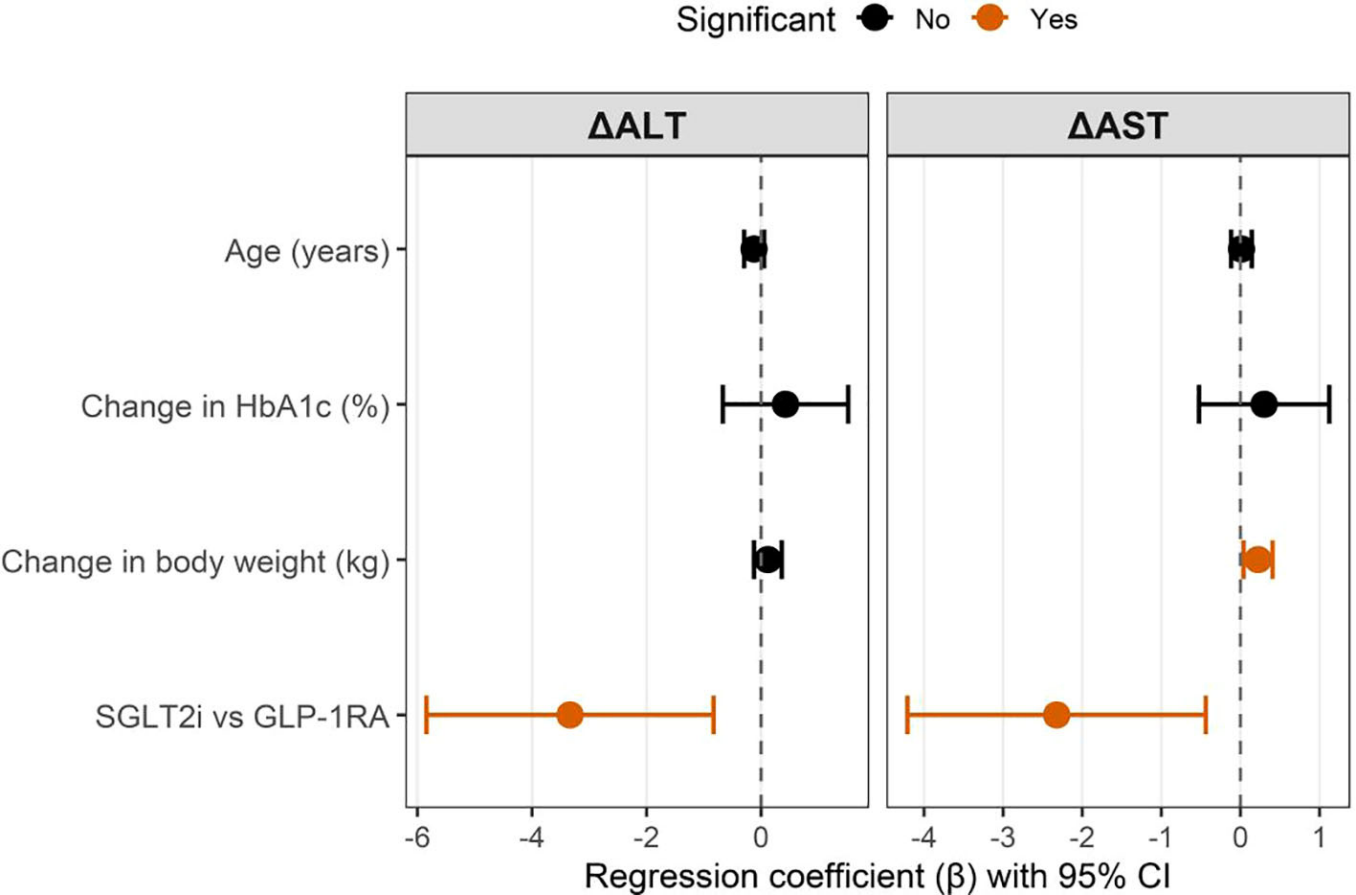
Forest plots of multivariable linear regression results for changes in ALT and AST after propensity score matching. GLP-1RA, glucagon-like peptide-1 receptor agonists; SGLT2i, sodium–glucose cotransporter 2 inhibitors; ALT, alanine aminotransferases; AST, aspartate aminotransferases; HbA1c, haemoglobin A1c.

A multivariable linear regression analysis was conducted in the matched cohort to isolate the independent effect of the therapeutic agent on changes in liver enzyme levels. With respect to ΔALT, following adjustment for baseline ALT, Δbody weight, ΔHbA1c, age, and sex, the treatment assignment (SGLT2is vs. GLP-1RAs) emerged as the sole independent determinant (β = -3.34, SE = 1.28, P = 0.009). In the model for ΔAST, both treatment assignment (β = -2.32, SE = 0.96, P = 0.016) and change in body weight (β = 0.22, SE = 0.09, P = 0.016) retained independent statistical significance (**Table 3**). In summary, the regression analyses substantiated that SGLT2is therapy was independently associated with more pronounced decreases in ALT and AST levels. Whereas body weight changes independently correlated with the reduction in AST, it was not independently associated with the change in ALT (**Figure 3**).

**Table 3:** Multivariable linear regression analyses for changes in ALT and AST after propensity score matching.

Variables	ΔALT (β ± SE)	P value	ΔAST (β ± SE)	P value
Treatment (SGLT2i vs GLP-1RA)	−3.34 ± 1.28	0.009*	−2.32 ± 0.96	0.016*
Baseline ALT/AST (U/L)	−0.08 ± 0.05	0.071	0.04 ± 0.05	0.391
Change in body weight (kg)	0.12 ± 0.12	0.338	0.22 ± 0.09	0.016*
Change in HbA1c (%)	0.43 ± 0.56	0.445	0.30 ± 0.42	0.474
Age (years)	−0.12 ± 0.09	0.172	0.01 ± 0.07	0.838
Sex (male vs female)	−0.37 ± 1.31	0.778	0.58 ± 0.98	0.555

Data are presented as regression coefficients (β) ± standard error (SE) with corresponding P values. *Indicates statistical significance. GLP-1RA, glucagon-like peptide-1 receptor agonists; SGLT2i, sodium–glucose cotransporter 2 inhibitors; ALT, alanine aminotransferases; AST, aspartate aminotransferases; HbA1c, haemoglobin A1c.

## 4 Discussion

This multicentre retrospective cohort study directly compared the effects of SGLT2is versus GLP-1RAs on liver function in patients with T2D and concomitant NAFLD. Results demonstrated that SGLT2is therapy significantly more effectively reduced ALT and AST levels compared with GLP-1RAs therapy. This difference remained statistically significant after rigorous propensity score matching to control for baseline confounding factors. Notably, multivariate regression analysis revealed that the significant reduction in ALT by SGLT2is was independent of weight change or glycaemic control, whereas the reduction in AST was associated with both SGLT2is treatment and weight loss. These findings provide new insights into the differential hepatic effects of these two widely used classes of antidiabetic medications.

Our findings extend the growing body of evidence supporting the hepatoprotective effects of SGLT2is in patients with metabolic dysfunction-associated liver disease. Bea et al., in a large nationwide cohort study from Korea, demonstrated that SGLT2is use was associated with a significantly lower risk of hepatic decompensation events compared to thiazolidinediones (HR 0.77, 95% CI 0.72-0.82), while showing similar effectiveness to GLP-1RAs (HR 0.93, 95% CI 0.76-1.14) (17). Although our study focused on liver enzyme changes rather than hard clinical outcomes, the consistency of findings—particularly the comparable efficacy between SGLT2is and GLP-1RAs—reinforces the credibility of both studies. Similarly, a recent network meta-analysis by Li et al. encompassing 737,408 patients with MASLD and diabetes identified GLP-1RAs and SGLT2is as the most effective glucose-lowering drugs for reducing liver-related events, with SUCRA values of 90% and 80%, respectively (18). Importantly, their pairwise meta-analysis revealed no significant difference between GLP-1RAs and SGLT2is for liver-related events (pooled HR 0.94, 95% CI 0.85-1.04), aligning closely with our observation of comparable but directionally favorable effects for SGLT2is on liver enzymes (18). The superior ALT reduction with SGLT2i observed in our study warrants particular attention. Several randomized controlled trials have previously demonstrated the efficacy of SGLT2is in reducing liver fat content and improving liver enzymes. The E-LIFT trial showed that empagliflozin significantly reduced ALT levels and liver fat content in patients with T2D and NAFLD (13). Similarly, Kahl et al. reported that empagliflozin effectively lowered liver fat content in well-controlled T2D (19). However, these studies lacked direct comparison with GLP-1RAs, which has also demonstrated substantial benefits in NAFLD. The LEAN trial established liraglutide’s efficacy in achieving resolution of non-alcoholic steatohepatitis (11), and subsequent studies with semaglutide have shown even more pronounced effects on liver fibrosis (12). Our head-to-head comparison thus addresses an important evidence gap by directly comparing these two drug classes under identical conditions.

One of the most intriguing findings of our study is the divergent pattern of association between treatment and the two liver enzymes. In multivariable regression analysis, SGLT2is treatment remained independently associated with ALT reduction after adjusting for weight loss and glycemic control, whereas AST reduction was independently associated with both SGLT2is treatment and weight loss. This dissociation may reflect the distinct biological characteristics of these enzymes and their differential responsiveness to direct hepatic versus systemic metabolic effects.

ALT is primarily localised in the cytoplasm of hepatocytes and is considered a more specific marker of hepatic cell damage (20). The lack of association between reduced ALT levels and weight loss suggests that SGLT2is may exert a direct protective effect on the liver. Multiple biological pathways may account for this direct effect. The hepatoprotective effects of SGLT2is are thought to be mediated through several complementary pathways, including: activation of AMP-activated protein kinase (AMPK), which in turn enhances fatty acid β-oxidation in the liver and inhibits *de novo* lipogenesis; alleviation of oxidative stress and endoplasmic reticulum stress; and inhibition of the NLRP3 inflammasome, thereby downregulating pro-inflammatory cytokines such as IL-1β and TNF-α (21). Research has shown that SGLT2is activate AMPK in liver cells; this pathway inhibits lipogenesis and enhances fatty acid oxidation (22). Li et al. demonstrated in animal models that dapagliflozin alleviates hepatic steatosis by restoring autophagy function via the AMPK-mTOR pathway (23). Furthermore, SGLT2is alleviate endoplasmic reticulum stress and inhibit NLRP3 inflammasomes, thereby mitigating hepatic inflammation and apoptosis (24). By modulating nutrient signalling pathways and enhancing renal uric acid excretion to lower serum uric acid levels, these agents also contribute to reducing hepatic lipid accumulation and improving insulin resistance (25, 26).

In contrast, GLP-1 receptor agonists primarily rely on weight-related mechanisms to improve hepatic steatosis, acting by slowing gastric emptying, suppressing appetite centrally and reducing calorie intake (27). However, recent evidence also supports the notion that GLP-1RAs exert direct effects on the liver: activation of hepatic GLP-1 receptors enhances hepatic insulin sensitivity, downregulates SREBP-1c expression (thereby reducing hepatic triglyceride synthesis), promotes autophagy-mediated clearance of lipid droplets, and induces the polarisation of hepatic macrophages from the pro-inflammatory M1 phenotype to the anti-inflammatory M2 phenotype (21, 28). GLP-1RAs have been shown to reduce *de novo* lipogenesis in the liver, lower oxidative stress and alleviate inflammation (29). In this study, the superiority of SGLT2is in improving ALT/AST levels may reflect a synergistic interaction between AMPK-dependent metabolic regulation and inflammasome-mediated anti-inflammatory effects; this hepatoprotective effect may have outweighed the primarily weight-dependent effects of GLP-1RAs during the six-month observation period of this study. It should be noted that in certain studies, GLP-1RAs have been shown to result in greater weight loss than SGLT2is (30), which may suggest that their hepatic benefits are more dependent on changes in body weight. Our study found that the reduction in ALT levels induced by SGLT2is was independent of body weight, whereas in previous studies, the reduction in ALT levels induced by GLP-1RAs was closely associated with weight loss (31), highlighting a potential mechanistic distinction between these two classes of drugs. As to whether GLP-1RAs can achieve hepatic benefits comparable to or even superior to those of SGLT2i over longer treatment periods (particularly when weight loss tends to plateau and histological improvements gradually become apparent), this remains to be verified by prospective extended follow-up studies.

Conversely, AST is localised in both cytoplasmic and mitochondrial compartments, expressed not only in the liver but also in cardiac, skeletal, and renal muscle (20). Its broader tissue distribution renders it more susceptible to systemic metabolic alterations. The independent association observed in this study between reduced AST and weight loss aligns with this understanding. Weight reduction improves systemic insulin sensitivity, alleviates adipose tissue inflammation, and decreases free fatty acid transport to the liver, thereby enhancing overall metabolic status (32). Consequently, the decrease in AST may reflect a combined effect of direct hepatic actions and systemic metabolic improvement, whereas ALT is more likely to specifically reflect direct hepatic effects.

The absence of significant differences in GGT changes between the two groups further supports this interpretation. GGT is more closely associated with oxidative stress and biliary tract function than with pure reflection of hepatocyte injury (33). The comparable reduction in GGT levels across both groups suggests these two classes of drugs may exert similar effects on oxidative stress pathways, whereas the divergence in ALT versus AST changes reflects their distinct mechanisms of action on hepatocyte integrity.

The findings of this study provide valuable guidance for clinical decision-making. For patients with T2D and NAFLD, particularly those with elevated ALT levels indicating active hepatocellular injury, SGLT2is may offer greater benefits than GLP-1RAs in terms of improving liver enzyme levels. When reducing liver enzyme levels is the primary treatment objective, clinicians may give priority to SGLT2is therapy. Furthermore, this effect is independent of weight loss. This finding may be particularly important for patients who struggle to achieve significant weight loss through lifestyle interventions. It should be emphasised that both classes of drugs have been shown to confer cardiovascular and renal benefits in large randomised controlled trials; therefore, the choice between them should still be guided by the principle of individualisation, taking into account the patient’s comorbidities (such as heart failure and chronic kidney disease), gastrointestinal tolerability, drug costs and patient preference.

Furthermore, our findings may have important clinical implications for patients with T2D and NAFLD who also present with complex cardiovascular and cerebrovascular disorders, such as Moyamoya disease. Moyamoya disease is a progressive, occlusive cerebrovascular illness characterized by bilateral stenosis of the terminal internal carotid arteries, leading to a highly fragile haemodynamic status that requires careful clinicopathological evaluation (34). A recent large-scale retrospective study by Roy et al. involving 2,898 T2D patients with Moyamoya disease demonstrated that the use of GLP-1RAs was associated with significantly reduced risks of stroke (HR 0.68), intracerebral haemorrhage (HR 0.35), and mortality (HR 0.32) (35). Additionally, while SGLT2is have been utilized in patients with Moyamoya disease, clinicians must remain vigilant regarding potential risks such as euglycemic diabetic ketoacidosis, given the complex vascular pathology of these patients (36). These findings suggest that in this highly vulnerable population, these medications offer cerebrovascular and systemic benefits extending beyond glycemic control. Although our study did not specifically include patients with such rare cerebrovascular complications, the observed hepatoprotective effects—particularly the weight-independent reduction of ALT by SGLT2is—further support the potential therapeutic value of these agents in T2D patients complicated by Moyamoya disease. Future prospective studies are warranted to fully delineate the risk-benefit profiles of these treatments in this specific patient cohort.

It should be acknowledged that this study has several limitations. Firstly, the study employed a retrospective observational design; even though propensity score matching was used to reduce measurable confounding, it is not possible to draw causal inferences. Unmeasured confounding factors, including concomitant medication (e.g. metformin, pioglitazone, vitamin E, statins), dietary habits, physical activity levels and baseline severity of liver fibrosis, may have introduced bias into the effect estimates. These residual confounders may have influenced the trajectories of liver enzyme changes in the two groups to varying degrees. Secondly, the identification of NAFLD relied on ICD-9/ICD-10 diagnostic codes, which may be subject to misclassification bias and do not reflect disease severity. The lack of histological data (liver biopsy) and quantitative imaging data (e.g., MRI-PDFF, FibroScan) limited our ability to assess the treatment’s impact on steatosis grading, lobular inflammation, hepatocyte ballooning, and fibrosis stage—which are precisely the core endpoints for evaluating disease-modifying efficacy in NAFLD. Future studies should incorporate paired biopsies or validated non-invasive imaging endpoints. Thirdly, whilst a 6-month follow-up period is sufficient to detect short-term changes in transaminases, it may be insufficient to capture longer-term histological improvements or clinical outcomes such as the progression of cirrhosis, liver decompensation, or hepatocellular carcinoma. Prospective studies with longer follow-up periods are required to continuously assess liver enzymes, non-invasive fibrosis markers and (where feasible) imaging or histological endpoints, in order to evaluate the durability and clinical significance of the observed biochemical improvements. Fourthly, the study participants were recruited from two medical centres in China, which may limit the generalisability of the findings to other populations and healthcare settings. Validation in multi-ethnic, multicentre cohorts is essential. Fifth, although ALT and AST are widely used and clinically accessible markers of hepatocyte injury, their sensitivity and specificity for NAFLD disease activity are suboptimal, and changes in transaminase levels do not necessarily correlate with histological improvement. The inclusion of more specific non-invasive biomarkers (such as cytokeratin 18 fragments and NAFLD fibrosis scores) would help to strengthen the evidence base in future studies.

## 5 Conclusion

In this multicentre retrospective cohort study of patients with T2D and NAFLD, SGLT2is therapy significantly reduced ALT and AST levels, with the ALT reduction being independent of weight loss. These findings suggest that SGLT2is may exert direct hepatoprotective effects beyond metabolic benefits, offering a valuable therapeutic option for patients with T2D and NAFLD, particularly when mitigating hepatocellular injury constitutes a clinical priority. However, given the observational nature of this study, these findings warrant further validation through large-scale randomised controlled trials.

## Data Availability

The raw data supporting the conclusions of this article will be made available by the authors, without undue reservation.
